# Differential Neuroprotective Effects of 5′-Deoxy-5′-Methylthioadenosine

**DOI:** 10.1371/journal.pone.0090671

**Published:** 2014-03-05

**Authors:** Beatriz Moreno, Iciar Lopez, Begoña Fernández-Díez, Miroslav Gottlieb, Carlos Matute, María Victoria Sánchez-Gómez, María Domercq, Albert Giralt, Jordi Alberch, Kevin W. Collon, Helen Zhang, Jack M. Parent, Meritxell Teixido, Ernest Giralt, Valentín Ceña, Inmaculada Posadas, Eva Martínez-Pinilla, Pablo Villoslada, Rafael Franco

**Affiliations:** 1 Center of Neuroimmunology, Institut d'investigacions Biomèdiques August Pi I Sunyer (IDIBAPS) - Hospital Clinic of Barcelona, Barcelona, Spain; 2 CIMA, Neurosciences Division, University of Navarra, Pamplona, Spain; 3 Oncology Area, Lung Cancer Unit, Center for Biomedical Research of la Rioja (CIBIR), Logroño, Spain; 4 Department of Neurosciences, University of Basque Country, Achucarro Basque Center for Neuroscience, Leioa, Spain; 5 Institute of Neurobiology, Slovak Academy of Sciences, Kosice, Slovak Republic; 6 Centro de Investigaciones Biomédicas en red en Enfermedades Neurodegenerativas (CIBERNED), Madrid, Spain; 7 Department of Cellular Biology, Immunology and Neurosciences, Medicine School, University of Barcelona, Barcelona, Spain; 8 Department of Neurology, University of Michigan Medical Center, Ann Arbor, Michigan, United States of America; 9 Institute for Research in Biomedicine (IRB), Barcelona, Spain; 10 Universidad de Castilla la Mancha, Albacete, Spain; 11 Department of Biochemistry and Molecular Biology, School of Biology, University of Barcelona, Barcelona, Spain; Charite Universitätsmedizin Berlin, Germany

## Abstract

**Background:**

5′-deoxy-5′-methylthioadenosine (MTA) is an endogenous compound produced through the metabolism of polyamines. The therapeutic potential of MTA has been assayed mainly in liver diseases and, more recently, in animal models of multiple sclerosis. The aim of this study was to determine the neuroprotective effect of this molecule *in vitro* and to assess whether MTA can cross the blood brain barrier (BBB) in order to also analyze its potential neuroprotective efficacy *in vivo*.

**Methods:**

Neuroprotection was assessed *in vitro* using models of excitotoxicity in primary neurons, mixed astrocyte-neuron and primary oligodendrocyte cultures. The capacity of MTA to cross the BBB was measured in an artificial membrane assay and using an *in vitro* cell model. Finally, *in vivo* tests were performed in models of hypoxic brain damage, Parkinson's disease and epilepsy.

**Results:**

MTA displays a wide array of neuroprotective activities against different insults *in vitro*. While the data from the two complementary approaches adopted indicate that MTA is likely to cross the BBB, the *in vivo* data showed that MTA may provide therapeutic benefits in specific circumstances. Whereas MTA reduced the neuronal cell death in pilocarpine-induced status epilepticus and the size of the lesion in global but not focal ischemic brain damage, it was ineffective in preserving dopaminergic neurons of the *substantia nigra* in the 1-methyl-4-phenyl-1,2,3,6-tetrahydro-pyridine (MPTP)-mice model. However, in this model of Parkinson's disease the combined administration of MTA and an A_2A_ adenosine receptor antagonist did produce significant neuroprotection in this brain region.

**Conclusion:**

MTA may potentially offer therapeutic neuroprotection.

## Introduction

The sulfur-containing nucleoside 5′-deoxy-5′-methylthioadenosine (MTA, CAS 2457-80-9) is produced from S-adenosylmethionine during the synthesis of the polyamines spermine and spermidine [1], and it acts as a potent inhibitor of polyamine biosynthesis. MTA is metabolized by MTA-phosphorylase to yield 5-methylthioribose-1-phosphate and adenine, a crucial step in the methionine and purine salvage pathways, respectively. The potential of this natural compound as a therapeutic agent was first demonstrated in experimental models of acute and chronic liver damage, and liver carcinogenesis, for which high doses were used without producing any significant toxicity [2]–[3]. More recently, MTA was proved to have a remarkable immunomodulatory activity in animal models of neuroinflammation, such as experimental autoimmune encephalomyelitis (EAE) [4]. In this model, when MTA was compared with other currently approved therapies for multiple sclerosis (MS), it was more effective and better suited for combination therapy [5].

The effects of inhibiting polyamine metabolism on brain function have not been addressed extensively, nor have the direct actions of MTA in the central nervous system (CNS) been investigated in detail. MTA was shown to have mixed effects on nerve growth factor (NGF)-regulated survival and protein phosphorylation in sympathetic neurons isolated from chick embryos, whereby MTA selectively blocked the NGF- but not high K^+^-mediated survival of neurons [6]. By assessing the changes in protein phosphorylation and methylation some early molecular events involved in NGF-mediated neuronal survival were seen to differ from those associated with high K^+^-mediated survival. Accordingly, MTA can block the NGF- but not the high K^+^-mediated decrease in phosphorylated p70, thereby showing that neural survival may involve distinct protein phosphorylation pathways, although these may later converge [6].

Clinical experiences with NGF suggest that neurotrophins or compounds with neurotrophin-like actions might be useful to develop new strategies to treat Parkinson's disease (PD) and/or other neurodegenerative disorders [7]. In the light of the anti-inflammatory effects of MTA, we set out to assess whether MTA is a neuroprotective agent *in vitro* and in animal models of diseases involving neuroinflammation, oxidative stress and excitotoxic damage, such as stroke, PD and epilepsy.

## Methods

### Animals

Experiments were performed in accordance with the Guidelines for the Care and Use of Mammals in Neuroscience and Behavioral Research (2003), and they were approved by the Ethical Committee for Animal Testing of the University of Navarra, University of Basque Country, University of Castilla la Mancha and University of Michigan, and by the Department of Health of the Government of Navarra. The animals were maintained in positive pressure-ventilated racks at 25±1° on a 12 h light/dark cycle and fed *ad libitum*.

### Primary cultures

Primary rat oligodendrocyte (OL) cultures were derived from the optic nerve of 12-day-old Sprague-Dawley rats, as described previously [8]. Cells were seeded onto 12 mm diameter coverslips coated with poly-D-lysine (10 µg/ml) in 24-well plates at a density of 5×10^3^ cells per well. The cells were maintained at 37°C in 5% CO_2_ and in a chemically defined medium [8]. After 2–4 days in vitro, the cultures were composed of at least 98% O4/galactocerebroside-positive (O4/GalC) cells, while the majority of the remaining cells were stained with antibodies against glial fibrillary acidic protein (GFAP). No A2B5^+^ or microglial cells were detected in these cultures [9].

Primary cultures of rat brain cortical neurons were prepared essentially as described previously [10]. The fronto-lateral cortical lobes were dissected out of Sprague-Dawley embryonic day 17 fetuses and they were dissociated mechanically in Hanks' balanced solution. After centrifugation at 800×*g* for 5 min, the cells were resuspended in serum-free Neurobasal medium (GIBCO) supplemented with B27 (GIBCO) containing 2 mM l-glutamine, penicillin (20 units/ml) and streptomycin (5 µg/ml), and they were plated onto poly-L-lysine-coated 24-well culture plates or on poly-L-lysine-coated 6-well culture plates. The cells were maintained at 37°C in a 95% air and 5% CO_2_ atmosphere of saturated humidity, and the cortical neurons were used for experiments after 7 days in vitro.

Finally, to prepare neuron-astrocyte co-cultures, neurons were resuspended in B27 Neurobasal medium plus 10% of foetal bovine serum (FBS) and then seeded onto a monolayer of astrocytes in 24-well plates at a density of 1–2×10^5^ cells per well, the astrocytes having been prepared beforehand, as described elsewhere [11]. One day later, the medium was replaced with B27-Neurobasal plus 10% FBS medium, and the cells were maintained at 37°C in 5% CO_2_. The cultures were used 8–9 days after plating. Neurons and astrocytes were identified with antibodies against microtubule-associated protein-2 and GFAP, respectively.

To assay N-methyl–D-aspartate (NMDA) excitotoxicity, primary cultures were seeded in 24-well plates at 15×10^4^ cells/well, cultured for 7 days in vitro, and treated for different times with the vehicle alone (dimethyl sulfoxide (DMSO) 1%), NMDA (300 µM), NMDA (300 µM) + MTA (250 µM) or NMDA (300 µM) + MK-801, a NMDA receptor antagonist (10 µM). The supernatants were collected and the cells were washed with PBS and lysed with 0.9% Triton X-100 (v/v) in saline. Caspase 3 activity was measured in these lysates one hour after the NMDA addition.

For a-amino-3-hydroxy-5-methyl-4-isoxazolepropionic acid (AMPA) assays in primary rat OLs, cells were pre-incubated with MTA (100 µM and 300 µM) for 15 min and then exposed to AMPA (10 µM) for an additional period of 15 min. Cell viability was assessed with calcein-AM (Invitrogen) 24 h after application of AMPA, and the number of survival cells on each coverslip was detected by their fluorescence (515 nm filter) and quantified.

### Oxygen-glucose deprivation (OGD) in primary rat cultures

Ischemic damage was induced by incubating cell cultures for 1 h in a hyperbaric chamber in a nitrogen-saturated buffer containing: 130 mM NaCl, 5.4 mM KCl, 0.1323 mM CaCl_2_, 0.26 mM NaHCO_3_, 0.8 mM MgCl_2_, 1.18 mM NaH_2_PO_4_, 10 mM sucrose (OGD plus sucrose) and iodoacetate (IAA, 20 or 50 µM). IAA is a glycolytic blocker typically used in OGD experiments to simulate ischemic conditions [12]. After the insult, the cells were incubated under normoxic conditions for an additional 24 h in the presence of glucose but in the absence of IAA. Distinct reagents were added during ischemia to evaluate their neuroprotective activity by assessing cell viability and cell death in the cultures.

### Cell viability

#### LDH assay

The cell status was assessed by measuring the release of lactate dehydrogenase (LDH) from dead cells. LDH was measured spectrophotometrically at 490 nm on a 96-well plate reader using the Cytotox 96 Kit according to the manufacturer's instructions (Promega). The LDH release was defined by the ratio of LDH released to the total LDH present in the cell at the beginning of the treatment.

#### Caspase 3 activity

Cortical neurons, or mixed cortical neurons and glia, were seeded in poly-L-lysine-coated 6-well culture plates during 7 days in vitro before start the set of experiments. In co-treatment conditions cells were treated for 1 h with NMDA (300 µM) alone or in the presence of MTA (100, 250 and 500 µM) or MK-801 (10 µM). In pre-treatment conditions MTA or MK-801 were added 15 minutes before NMDA. To determine caspase 3 activity, the cells were washed twice with cold PBS and lysed in buffer containing 100 mM HEPES, 5 mM dithiothreitol (DTT), 5 mM ethylene glycol tetra-acetic acid (EGTA), 0.04% Nonidet P-40 and 20% glycerol (pH 7.4). The extracts were then centrifuged at 5,000×*g* for 10 min at 4°C and the protein content was determined by the BCA protein assay. Cell extracts (40 µg of protein) were incubated for 1 h at 37°C in a reaction buffer (25 mM HEPES, 10% sucrose, 0.1% CHAPS, 10 mM DTT) containing 50 µM of the fluorescence substrate, Z-DEVD-AFC. Cleavage of the AFC fluorophore was determined in a spectrofluorometer at an excitation wavelength of 400 nm and the fluorescence emitted was detected at a wavelength of 505 nm. Caspase 3 activity was expressed as units of fluorescence per milligram of protein per hour.

### Animal models of disease

#### Focal and global ischemia models in rats

Male Wistar rats (270-300 g; Harlan) were subjected to transient focal ischemia for 90 min by producing right middle cerebral artery occlusion (MCAO) as described previously [13], followed by reperfusion for 3 days and MTA treatment (30 mg/kg, intraperitoneally –i.p.- twice daily) starting 30 min after the onset of ischemia (n = 5 in each experimental group). Cerebral blood flow was monitored transcranially with a laser Doppler flowmeter (Perimed) and the infarct volume was assessed by 2,3,5-triphenyltetrazolium chloride (TTC) staining. Transient forebrain ischemia was induced as described elsewhere [14], by occluding the vertebral and common carotid arteries (4VO) for 10 min, followed by reperfusion for 7 days and MTA treatment (as above) starting 30 min after the onset of ischemia (n = 5 in vehicle group; n = 6 in the vehicle group). The effectiveness of the treatment was assessed by using fluoro-Jade C staining to measure the levels of degenerated neurons in the CA1 region of the hippocampal formation.

#### Status epilepticus rat model

Young adult (175–200 g) male Sprague-Dawley rats from Charles River (Wilmington, MA, US) were divided into four MTA treatment groups, with 9–11 rats per group. In group A, rats were subjected to pilocarpine-induced status epilepticus (SE) as described previously [15]. Following 90 min of continuous seizure activity, SE was stopped by administering a single dose of diazepam (10 mg/kg). The rats were then injected daily for 3 days with either the vehicle alone (2% DMSO in H_2_O), injecting a volume of body weight x 3.3 (a similar volume as for the MTA treated groups), or MTA (30 mg/kg). Rats were euthanized on day 4 after SE under anesthesia by transcardiac perfusion with 4% paraformaldehyde (PFA) in phosphate-buffered saline. The brains were removed, postfixed in 4% PFA, cryoprotected in 30% sucrose, frozen, and sectioned for histology. In group B, the rats received the same treatment as those in group A (vehicle or MTA 30 mg/kg) except that the rats were perfused on day 30 after SE and a 0,37% sulphide fixative was used for subsequent Timm staining. In group C, the rats were only treated with MTA (30 mg/kg) or the vehicle for 2 days and on the third day, the rats were again subjected to pilocarpine-induced SE. The animals were then treated once daily with with MTA (30 mg/kg) or vehicle for a further 3 days, and they were anesthetized and euthanized 4 days after SE. In group D, the rats received the same treatment as group C (vehicle or MTA 30 mg/kg) except that they were euthanized 30 days after SE and a 0,37% suphide fixative was also used foe subsequent Timm staining. The brain sections obtained from these animals were stained with cresyl violet (Nissl), Timm Stain or by immunohistochemistry for Neu N.

#### MPTP mouse model of Parkinson's disease

12-week-old male C57Bl mice from Charles River (Barcelona, Spain) were injected i.p. with 30 mg/kg 1-methyl-4-phenyl-1,2,3,6-tetrahydropyridine (MPTP, Sigma-Aldrich) on five consecutive days. Subsequently, MTA (30 mg/kg) and/or the A_2A_ adenosine receptor (A_2A_R, 10 mg/kg) antagonist, MSX-3 (Sigma-Aldrich), was administered one day before starting MPTP treatment and 1 h after each MPTP injection (n = 6 in each experimental group). We used mice in the MPTP model because rats are resistant to systemic MPTP administration and thus, the rat MPTP model requires stereotaxic MPTP injection directly into the nigrostriatal fibers. Systemic administration destroys adrenergic neurons of sympathetic ganglia but it is not toxic in the CNS.

### Tissue processing

Animals were anesthetized with an overdose of 10% chloral hydrate (Panreac, Barcelona, Spain) in distilled water and perfused transcardially with a fixative solution containing 4% PFA (Merck, Darmstadt, Germany) in 0.125 M PBS. The brain of each animal was removed and cryoprotected for 48 h in PBS containing 20% glycerin and 2% DMSO. All solutions used for perfusion and cryoprotection were treated with 0.1% diethylpyrocarbonate (DEPC) and autoclaved prior to use. Frozen coronal microtome sections (50 µm thick) were collected in cryoprotective solution and stored at −80°.

### Fluoro-Jade C staining

Fluoro-Jade C (Chemicon, Millipore) was used to visualize dying neurons in 10 µm frozen coronal sections pretreated for 15 min with 0.06% KMnO_4_. After washing in distilled water, the sections were incubated for 30 min in fluoro-Jade (0.0001% in 0.1% acetic acid), washed again, dried, cleared in xylene and coverslipped with DPX. Serial photomicrographs of the hippocampal CA1 regions were collected at random with a digital camera using a 20× objective. Cells were counted in the right and left hemispheres of each stained section, examining four sections from each experiment.

### Nissl and Timm staining

Mouse and rat coronal sections were mounted on glass slides in a 0.2% solution of gelatin in 0.05 M Trizma base [pH 7.6] (Sigma-Aldrich), and they were dried overnight at room temperature (RT). Following incubation with a mixture of chloroform and ethanol for 1 h, the sections were hydrated serially in 100%, 96%, 80% and 50% alcohol, and rinsed twice in distilled water for 2 min. Nissl staining was performed for 2 min with a solution containing 8.7 mM thionine (Sigma-Aldrich), 30 mM NaOH (Sigma-Aldrich) and 1.2% ethanol. The sections were then washed with distilled water, dehydrated with successive 2 min incubations in increasing concentrations of alcohol (70, 96 and 100%), cleared in xylene for 6 min and cover slipped with DPX (BDH Chemicals). For Timm staining, sections were mounted onto superfrost plus slides (Fisher, Pittsburgh, PA) and then dehydrated, rehydrated and placed in a staining solution containing gum arabic, citrate, hydroquinone and silver nitrate and incubated at 26°C. After development of the stain, sections were washed, dehydrated and delipidated in graded ethanols and xylenes, and coverslipped. To evaluate sprouting of the zinc-containing mossy fibers in the dentate gyrus inner molecular layer, Timm-stained sections were analyzed by an examiner blinded to experimental condition. A minimum of 5 sections, spanning the rostral, mid, and caudal dentate gyrus were examined by densitometry. Twelve samples of pixel density/section were measured in the inner molecular layer using NIH ImageJ, 5 from the lower blade, 5 from the upper blade and 2 from the apex. The average background level (mean of 12 samples/section) was subtracted and a mean pixel density calculated for each animal (n = 4–5/group).

### Immunohistochemistry

Free-floating coronal brain sections were washed sequentially with PBS, treated with 0.03% H_2_O_2_ in methanol for 40 min to block endogenous peroxidase activity and washed a further three times with PBS. Non-specific binding was blocked by incubating for 40 min at RT in a blocking solution: 4% normal goat serum, 0.05% Triton X-100 and 4% BSA in PBS. The sections were incubated overnight at 4° with a specific anti-tyrosine hydroxylase (TH) antibody (1∶500; clone 16 T-2928 Sigma-Aldrich) and with mouse monoclonal anti-NeuN (1∶1000; Millipore, Billerica, MA, US), both diluted in blocking solution. After washing with PBS several times, the sections were incubated for 2 h at RT with the corresponding secondary antibody diluted in blocking solution, a biotinylated goat anti-rabbit or biotinylated goat anti-mouse (1∶300; Jackson ImmunoResearch Laboratories, West Grove, PA). After washing several times with PBS, the sections were incubated for 90 min with streptavidin-HRP (1∶4000; Sigma-Aldrich) and the bound peroxidase activity was visualized by incubation with 0.05% diaminobenzidine (Sigma-Aldrich) in 0.03% H_2_O_2_/Tris-HCl [pH 7.6]. The sections were mounted on glass slides in a 0.2% solution of gelatin in 0.05 M Tris [pH 7.6] (Sigma-Aldrich), dried overnight and dehydrated in toluene for 12 min before they were coverslipped with DPX (BDH Chemicals, Poole, UK). The usual tests to control for specificity were performed in parallel.

### Stereological counting

In the pilocarpine SE model, NeuN-positive cells in the hippocampal CA1 and CA3 regions were counted stereologically in the 30-day survival groups. The regions of interest were outlined at low magnification (4×) and the NeuN-labeled cells were counted at 100× using the optical fractionator technique (StereoInvestigator software, MicroBrightField) with systematic random sampling of every 6th section in a series of 50 µm coronal sections through the hippocampus.

In the MPTP mouse model, the substantia nigra *pars compacta* (SNpc) volume was estimated according to the Cavalieri method. The number of TH-positive neurons was established through unbiased design-based stereology, performed blind to the genotype and group. All stereological counting was performed with the newCAST Visiopharm (Denmark) software using an Olympus Bx61 microscope equipped with a camera (model DP71, Olympus) and a stage connected to a xyz stepper (model H101BX, PRIOR). Specifically, we used the dissector counting procedure in six SNpc coronal sections (50 µm thick) per animal, equally-spaced and covering the entire rostro-caudal extent of the nucleus.

The reference volume was calculated according to Cavalieri principles from images obtained with a 2× objective using a point count array [16]. The cross-sectional area of the nucleus was measured and the reference volume (*Vr*) for the entire nucleus was estimated using the following equation:
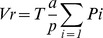
where T is the thickness of the sections, a/p is the area of each point and Pi is the number of points that landed within the area. The region of interest to estimate the area was outlined at low magnification (4×), and the number of labeled neurons was calculated under 100× magnification (with oil immersion) using randomized meander sampling and the optical dissector methods. The optical dissector height was 12 µm. The sampling area and the counting frame were 3% and 3,025.6 µm2, and unbiased counting was performed by an experimenter blind to the treatments. Total Nissl stained neurons (N) were calculated using the following formula:
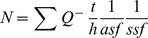
where ΣQ^−^ is the total number of particles counted, *t* is the mean section thickness, *h* is the height of the optical dissector, *asf* is the area sampling fraction, and *ssf* is the section sampling fraction. Neuronal density (*D*) was determined by the following formula: *D* =  *N*/*Vr.*

### Parallel artificial membrane permeability assay (PAMPA)

The PAMPA assay was used to determine the capacity of compounds to cross a blood brain barrier (BBB) model by passive diffusion, measuring the effective permeability of the compounds at an initial concentration of 200 µM. The buffer solution was prepared from a concentrated commercial solution (Pion, Billerica, MA, US) according to the manufacturer's instructions and the pH was adjusted to 7.4 with 0.5 M NaOH. The compound of interest was dissolved in the buffer solution (200 µM), the PAMPA sandwich was separated and the donor well was filled with 195 µL of the compound of interest. The acceptor plate and donor plate were brought together, with the underside of the membrane in contact with the buffer. An 4 µL aliquot of the phospholipid mixture (20 mg/ml) in dodecane was added to the filter of each well, and then 200 µL of buffer solution was added to each acceptor well. The plate was covered and incubated for 4 h at RT in a saturated humidity atmosphere with agitation and with 25 µm of an unstirred water layer in the Gut-Box (Pion). After 4 h, a 150 µL/well sample from the donor plate and a 150 µL/well sample from the acceptor plate were transferred to HPLC vials. Then, 100 µL from acceptor well, 5 µL from the donor well and 5 µL from the t_0_ samples were injected into an HPLC reverse-phase symmetry C18 column (150 mm×4.6 mm×5 µm, 100 Å; Waters). Transport was confirmed by HPLC mass spectrometry and matrix-assisted laser desorption/ionization time-of-flight (MALDI-TOF) spectrometry to ensure the compound had maintained its integrity. The phospholipid mixture used was a porcine polar brain lipid extract composed of 12.6% phosphatidylcholine (PC), 33.1% phosphatidylethanolamine (PE), 18.5% phosphatidylserine (PS), 4.1% phosphatidylinositol (PI), 0.8% phosphatidic acid and 30.9% of other compounds.

### *In vitro* model of transport across the BBB

A *in vitro* cell model of BBB permeability was established using a co-culture of bovine-brain endothelial cells (BBECs) and newborn rat astrocytes. The cells were first cultured in 24-well (Transwell^R^) permeable supports with a surface area of 0.33 cm^2^ and a pore size of 0.4 µm (Corning), the upper surface of which was coated with collagen type IV and fibronectin. The inserts were then placed upside down in a large petri dish, and 40 µL of a suspension (containing approximately 45,000 astrocytes) was placed on the bottom of each filter. The petri dish was incubated at 37°C for 1 h, and 40 µL of fresh DMEM+S was added to the bottom of each filter every 15 min. The inserts were then transferred back to the plate and incubated at 37°C in 5% CO_2_ for 3 d. Then, 2 h before seeding the BBECs, the medium was replaced by DMEM+S supplemented with 125 µg/mL heparin and 2 h later, the cells were seeded onto the inserts (45,000 cells per filter). The plate was incubated at 37°C in 5% CO_2_ for 3 more days, after which the medium was replaced by DMEM+S supplemented with cyclic adenosine monophosphate (cAMP) and RO-20-1724, and kept at 37°C in 5% CO_2_. On day 8 of the co-culture, the transendothelial electrical resistance (TEER) was measured to determine whether the system was ready for transport studies. To validate the maturity of the model, permeability assays with lucifer yellow (LY) were performed in parallel as a marker of the integrity of the in vitro barrier. During the permeability assay, the samples were co-incubated with LY (20 µM) to assess the integrity of the cellular monolayer during the assay.

### Data analysis

The data in the graphs are presented as the mean ± SEM and statistical analysis was performed with SPSS 18.0 software. Significance was tested with a Student *t*-test or one-way ANOVA test followed by a LSD, Bonferroni or Tukey HSD test as a post hoc. Significant differences were considered when *p*<0.05.

## Results

### MTA as a neuroprotective agent for neural cells cultured in vitro

**Figure 1:**
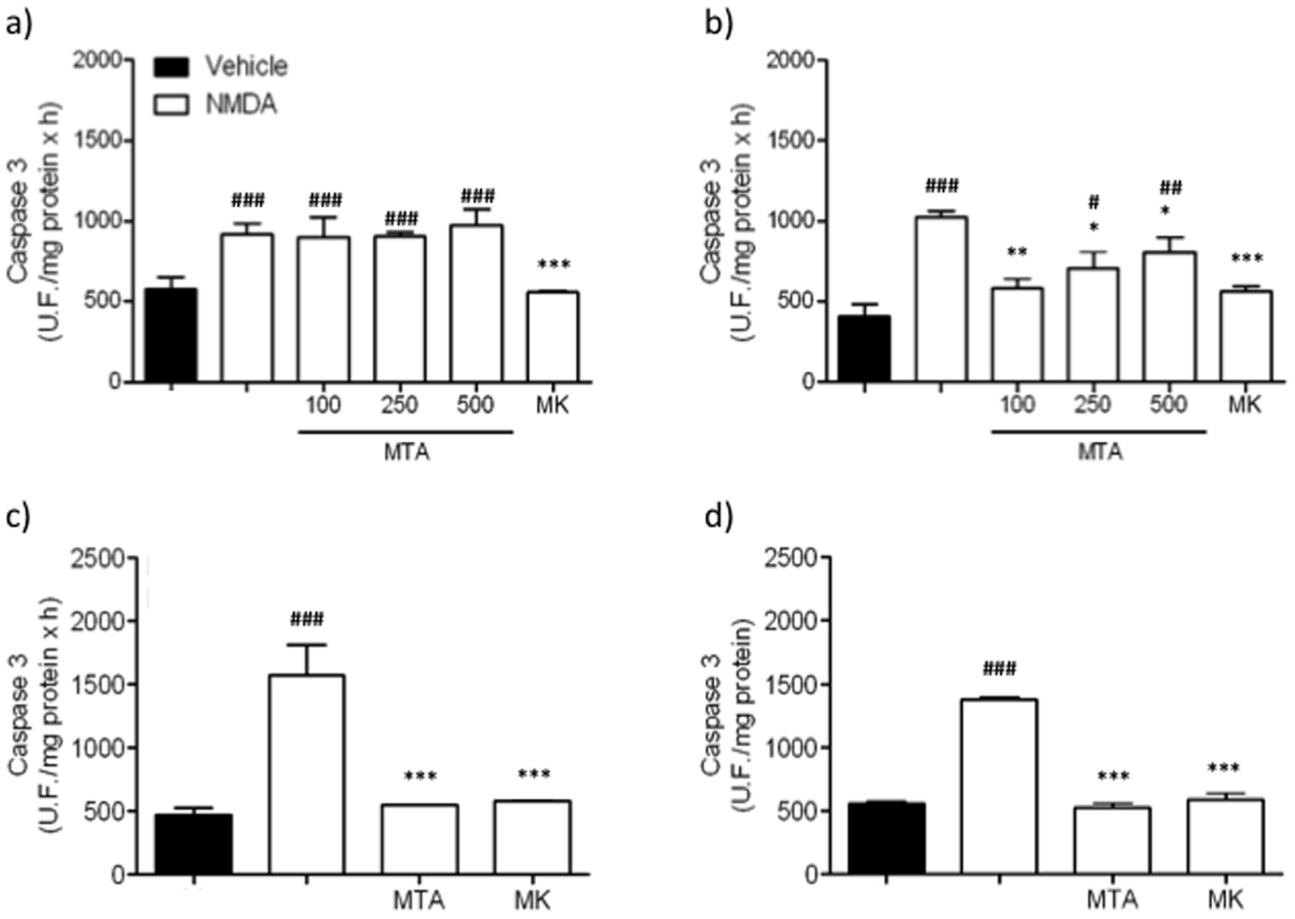
Effect of MTA under NMDA excitotoxicity. Effect of MTA co-treatment (a) or MTA pre-treatment (b) on NMDA-induced caspase 3 activation in rat pure primary neuronal cultures. Effect of MTA co-treatment (c) or MTA pre-treatment (d) on NMDA-induced caspase 3 activation in rat mixed astrocyte-neuron cultures. Caspase 3 activity (units of fluorescence per milligram of protein per hour) was determined in cells treated with 300 µM NMDA, in the presence or absence of MTA (panels a and b: 100, 250 and 500 µM; panels c and d: 250 µM) or 10 µM MK-801 (MK; NMDA receptor antagonist). The results are expressed as the mean ± SEM of at least four independent experiments performed in triplicate: *p<0.05, **p<0.01***p <0.001 compared with cells treated with NMDA; # *p*<0.05, ## *p*<0.01, ### *p*<0.001 compared with vehicle cells. One-way analysis of variance (ANOVA) and Bonferroni's t-test for multiple comparisons.

**Figure 2:**
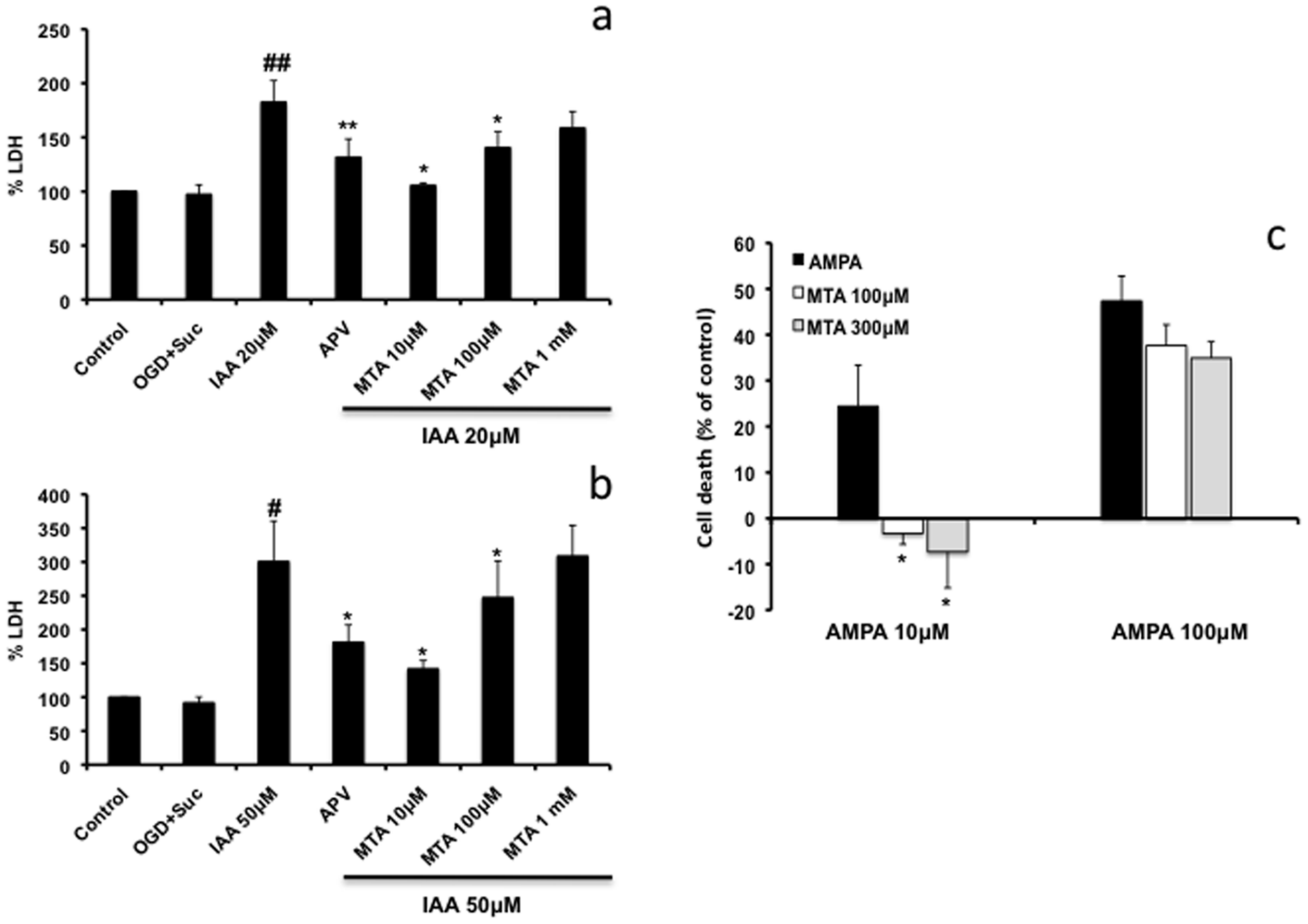
Effect of MTA under OGD and AMPA excitotoxicity. Panels a and b: the effect of APV (100 µM) or MTA in OGD conditions in rat mixed cultures of neurons and astrocytes. Cell death was expressed as the LDH activity at the beginning of any treatment: *p<0.05 compared to IAA-treated cells; # *p*<0.05 compared to control; ** *p*<0.01 compared to IAA-treated cells; ## *p*<0.01 compared to control. **c**) primary oligodendrocytes derived from rat optic nerve subjected to AMPA excitotoxicity (10 µM and 100 µM) in which cell death was measured with calcein-AM, as indicated in Methods. *p<0.05 compared with cells treated with AMPA (Unpaired t-test). Values represent the average ± SEM and were obtained from at least three independent experiments performed in duplicates.

To evaluate the neuroprotective potential of MTA at the cellular level, various cell models and noxious insults were used. Excitotoxicity seems to be involved in the pathogenesis of epilepsy, brain ischemia and other neurodegenerative diseases [17]. Accordingly, the role of MTA in promoting the survival of neurons treated with NMDA was tested. The survival of rat neurons in culture (99% purity) when exposed to toxic concentrations of NMDA (up to 500 µM) was not enhanced by co-treatment with MTA (as measured by caspase 3 activity: Fig. 1a). However, when neuronal cultures were pre-treated with MTA, caspase 3 activity decreased significantly at the different concentration tested (Figure 1b). When mixed cultures (neurons and astrocytes) were pre- or co-treated with MTA, we found that caspase 3 activation induced by NMDA reverted to basal levels, an effect also produced by the ionotropic glutamate receptor antagonist, MK-801 (Fig. 1c and 1d). These results suggest a key role for astrocytes in mediating some of the beneficial effects of MTA. The role of MTA in promoting survival was also tested in mixed rat neuronal-astrocyte cultures subjected to oxygen-glucose deprivation induced by IAA. At the two doses of IAA used (20 and 50 µM), protection afforded by 10 µM or 100 µM MTA was significant and similar to that provided by 100 µM of the NMDA receptor antagonist, (2R)-amino-5-phosphonovaleric acid (APV: Fig. 2 a–b). Interestingly, a supramaximal dose of MTA (1 mM) was neither protective nor did it lead to greater damage than OGD itself, suggesting that an excess of the compound does not exacerbate toxicity. Primary rat OL cultures were also treated with toxic doses of AMPA and cell activity was measured with calcein-AM, a probe that provides robust data in these oligodendrocyte cultures. It should be noted that OLs are more sensitive to AMPA than to NMDA excitotoxicity [18]. MTA fully protected OLs against AMPA toxicity (Figure 2c). These results indicate that MTA may protect neurons and oligodendrocytes from excitotoxic insults and that excess MTA does not enhance toxicity of neurons in pure or mixed neuron-glia cultures.

### MTA neuroprotection in animal models of ischemia, neurodegeneration and excitotoxicity

To evaluate the potential neuroprotection offered by MTA, we assessed its effects in animal models of brain diseases that involve inflammation and excitotoxicity. Any drug aimed at targeting neural cells in the CNS must cross the BBB. Therefore, we first measured the ability of MTA to cross the BBB *in vitro* using a PAMPA and an *in vitro* cell model of the BBB. In the PAMPA model, we found that MTA could cross the BBB by passive diffusion with a low to intermediate effective permeability as compared to the reference compounds, propranolol and carbamazepine (Table 1). Membrane retention of MTA was negligible, whereas it was significant for propranolol (30%: Table 1). The potential of MTA to cross the BBB was confirmed in an *in vitro* cell model, in which MTA displayed an effective permeability coefficient (Pe = 4.5×10^−6^ cm/s) in between that of the control compounds, transferrin (Pe  =  8.2×10^−6^ cm/s) and ApoE (Pe  =  2.2×10^−6^ cm/s: Table 1).

**Table 1:** Effective permeability (Pe) values in the PAMPA assay and in the *in vitro* model of BBB.

Compound	Model	% Membrane retention	Pe x 10^−6^ (cm/s)
Methylthioadenosine (MTA)	PAMPA	0	0.15*
Carbamazepine	PAMPA	0.5	11.5
Propanolol	PAMPA	30.2	10.3
Methylthioadenosine (MTA)	BBB	-----	4.5
Transferrin	BBB	-----	8.2
Apo E	BBB	-----	2.2

*transport confirmed by HPLC-MS.

**Figure 3:**
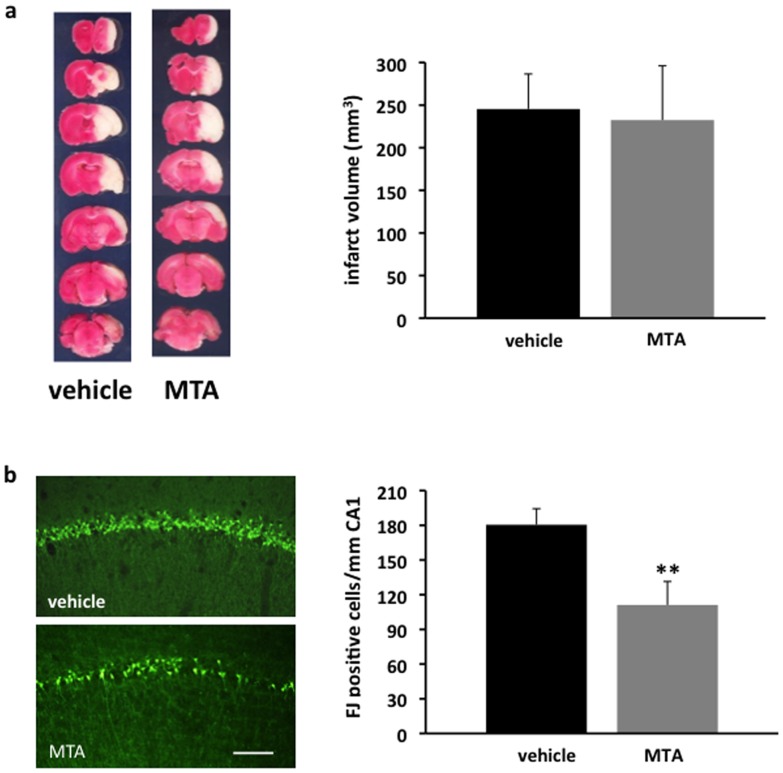
Effect of MTA on brain ischemia. (a) Representative TTC stained sections of vehicle and MTA treated animals (30 mg/kg/twice daily, i.p.) 3 days after the induction of transient focal ischemia. Histogram (right) showing the infarct volume calculated from TTC stained slices in vehicle- and MTA-treated rats (n = 5 in each group). b) Representative microphotographs of Fluoro Jade C staining after rat transient forebrain ischemia (n = 5 in the vehicle group; n = 6 in the MTA-treated group). MTA (30 mg/kg) was administered 30 min after triggering ischemia. Quantification of Fluoro Jade positive cells per mm length of CA1 pyramidal layer (right). The data represents the mean ± SEM: **p<0.01 compared to the vehicle (Student t-test). Scale bar 100 µm.

The neuroprotective effect of MTA was first evaluated in the rat transient MCAO model of focal brain ischemia. In this model, rats treated with MTA did not show any reduction in the volume of the infarct area compared with those treated with the vehicle alone (Fig. 3a). By contrast, MTA afforded protection to neurons following global brain ischemia, as fewer cells in the hippocampal CA1 region were stained with Fluoro Jade C following MTA administration (110±20 cells/mm) compared to those in untreated animals (180±10 cells/mm: Fig. 3b).

**Figure 4:**
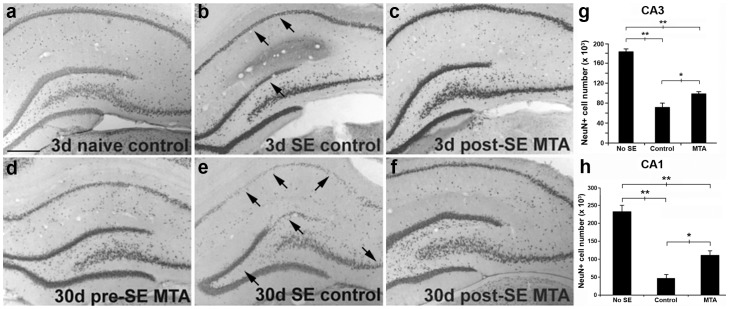
MTA effects in chronic pilocarpine-induced status epilepticus (SE). a–f: Representative images of Neu N immunoreactivity in the hippocampus are shown 3 days after sham treatment (a) or pilocarpine-induced SE (b, c), or 30 days after SE (d–f). MTA (30 mg/kg) was administered pre-SE (d) or post-SE (c,f) induction. Cell loss is already apparent by 3 days (arrows in b) and is marked by 30 days (e) after SE, but it appears attenuated in MTA-treated animals at both timepoints (c,d,f). g–h) Bar plots show quantification of NeuN-positive cells in CA3(g) or CA1(h) 30 days after SE in animals pre-treated with MTA. The data represent the mean ± SEM. *p<0.05; **p<0.001; ANOVA with Tukey HSD post –hoc test.

Temporal lobe epilepsy is associated with substantial neuronal loss in the hippocampus due to excitotoxicity and thus, we examined the effects of MTA (30 mg/kg/day) in the pilocarpine model of SE. Accordingly, we administered MTA for 5 days to one group of rats, beginning 2 days before SE (pre-SE MTA), while another group was given MTA for 3 days beginning at the termination of SE (post-SE MTA). Subsequently, brain sections were examined for cell loss either 3-5 days (early time point) or 30 days (late time point) after SE. As controls, one group received the vehicle alone instead of MTA and underwent SE, while a second control group received MTA and saline instead of pilocarpine (No SE). The SE model caused neuronal loss in the hippocampus, mainly in the CA1 and CA3 regions, and *dentate gyrus*, whereby these cell layers became thinner (Figure 4). The alterations in hippocampal structure displayed by these animals were more evident 30 days after SE (Fig. 4e, arrows). The administration of MTA either before or after SE reduced the neurodegeneration and neuronal loss observed in the aforementioned hippocampal areas at both the early and late time points analyzed, as witnessed by NeuN immunoreactivity (Fig. 4c, f). Indeed, 30 days after SE there were significant reductions in the neuronal loss in the CA3 and CA1 hippocampal areas of animals that received MTA prior to SE, as quantified through the unbiased stereological counting of NeuN-immunoreactive cells, althought they still showed significant loss compared to sham animals (Fig. 4g,h). No difference in mossy fiber sprouting was observed in either of the MTA treatment groups studied (Figure S1).

**Figure 5:**
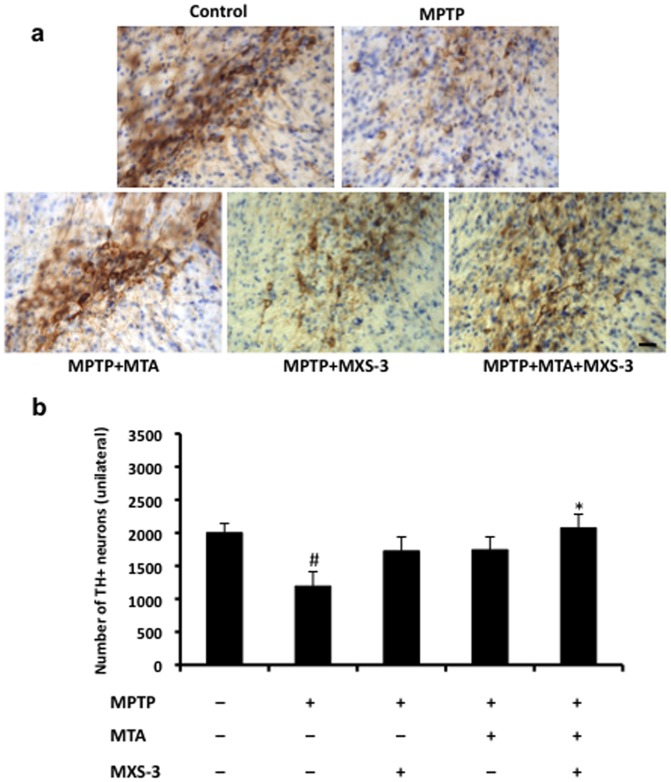
Effect of MTA and A_2A_R antagonists in an acute mouse model of PD. The effect of MTA (30 mg/kg), MSX-3 (9 mg/kg) or both in combination on the survival of dopaminergic neurons (TH^+^ cells) within the *substantia nigra* of the brain of mice treated with MPTP. a) Representative images of the neurodegeneration of dopaminergic cells in the different groups. b) Stereological counts of TH^+^ neurons in control and drug-treated animals (n = 6 per group). The data represent the mean±SEM: *p<0.05 comparing with the MPTP group; # *p*<0.05 comparing with the control group (One-way ANOVA, LSD test as a post-hoc). Scale bar: 50 µm.

In PD, the degeneration of dopaminergic neurons in the *substantia nigra* is in part due to oxidative stress and excitotoxicity. To assess whether MTA protects dopaminergic neurons from oxidative stress, we used acute MPTP administration since this neurotoxin produces in acute treatments very marked dopamine nigral denervation and dopaminergic neuronal loss that can be assessed by TH immunostaining (Fig. 5a). An A_2A_ adenosine receptor (A_2A_R) prodrug antagonist was also administered in this model as several A_2A_R antagonists are undergoing clinical trials to assess their capacity to alleviate the motor symptoms of PD and/or to prevent levodopa-induced involuntary movements. As such, MTA, the A_2A_R antagonist prodrug, MSX-3, or both were injected 24 h before MPTP and, on the next 4 days MTA, MSX-3 or both were injected 1 h before administering MPTP. Neuroprotection was quantified by unbiased stereological counting of TH^+^ neurons in the *substantia nigra*, which highlighted the neuroprotective trend of the individual treatments that was, nevertheless, not statistically significant. However, the combined treatment did lead to significant neuroprotection (Fig. 5b).

In summary, *in vivo* therapy with MTA in animal models of brain damage may protect neurons from death depending on the noxious stimulus and/or on combined administration with other drugs.

## Discussion

In this study we evaluated the ability of the natural metabolite MTA to protect the CNS from damage in cellular and animal models of neurological disease. Intermediate metabolism plays a critical role in maintaining cell homeostasis and in the responses to stress, as evident through the potentially beneficial anti-oxidant properties of fumaric acid, an intermediate metabolite of the Kreb's cycle, [19]. Here, we report the differential neuroprotective effects of MTA in different models of CNS disease.

In PD, apart from nigral neurodegeneration, extranigral neuropathological changes occur that include an extensive α-synuclein pathology, as well as neuroinflammatory responses in specific brain regions ([20]–[21] for review). In normal circumstances, only a few T lymphocytes are found circulating in the neuronal CNS parenchyma, reflecting their limited passage into the brain. However, the release of proinflammatory cytokines by microglia in PD-related conditions upregulates the expression of intercellular adhesion molecule-1 (ICAM-1) and vascular cell adhesion molecule-1 (VCAM-1) by endothelial cells of the BBB, which are counter receptors for cell surface proteins in T cells and monocytes [22]. Selective T cell infiltration of degenerative lesions reflects the changes in the parenchymal milieu that resemble those occurring in peripheral inflammatory lesions [23]. T lymphocytes contribute to neurodegeneration by amplifying and exacerbating ongoing inflammatory processes. Thus, both adaptive and innate immune responses appear to play a pivotal role in the pathogenesis of PD, providing a meaningful rationale for therapies that target the immune system [21]. MTA has previously been shown to provide benefits in animal models of MS, a disease that has a clear neuroinflammatory component [5]–[6]. In our study, it does not appear to protect against the neuroinflammation produced by MPTP in the PD mouse model when tested alone. However, when combined with an A_2A_ adenosine receptor antagonist, MTA therapy does result in neuroprotection, which is particularly significant given that A_2A_ adenosine receptor antagonists are currently being assessed in clinical trials for the symptomatic treatment of PD and the prevention of L-DOPA-induced dyskinesias. Indeed, these molecules are not considered to protect against the death of dopaminergic neurons in the course of PD. However, we show here that the combination of these two compounds, which individually are not efficacious, protects nigral neurons exposed to MPTP from damage. The anti-PD effect of L-DOPA, which is converted into dopamine, is exacerbated by A_2A_ receptor antagonists, as they decrease the negative adenosinergic tone and affect the function of postsynaptic D_2_ dopamine receptors. The protection provided by combined MTA and MSX-3 treatment is achieved presynaptically and it probably reflects the synergy between MTA-selective activities and the blockade of A_2A_ receptor signaling in the body of dopaminergic neurons within the *substantia nigra*.

The results reported here also indicate that MTA produces a marked preservation of CA1 and CA3 hippocampal neurons that are sensitive to pilocarpine, thereby raising the possibility of using MTA in the treatment of certain types of epilepsy. This hypothesis is further supported by the protection afforded in the cells subjected to excitotoxicity. Furthermore, we demonstrate the therapeutic potential of MTA in some aspects of hypoxia, both in cellular and animal models. MTA was effective in global ischemia but it failed to reduce the infarct area in the MCAO model of focal brain ischemia, two pathologically distinct events. While in focal ischemia the main damage is provoked by necrosis in the “ischemic core”, the global ischemia model diminishes blood flow over the entire brain, provoking neuronal death in more vulnerable areas, such as the CA1 of the hippocampus. These differences in the mechanisms that provoke damage and the distinct vulnerabilities of specific cell populations could explain the different effects of MTA on focal and global cerebral ischemia.

The mechanisms by which MTA mediates cell protection are complex and pleiotropic. The *in vitro* effects of therapies based on this endogenous metabolite involve its anti-oxidant capabilities [24], yet MTA is likely to have other effects *in vivo* as it influences numerous events that regulate gene expression, cell proliferation and differentiation, and apoptosis [2], [4]. Accordingly, it is reasonable to assume that an increased load of MTA alters secondary metabolism and has an impact on mechanisms involved in coping with oxidative stress, as well as on cell survival/death [25]. Indeed, pharmacological manipulation of other endogenous metabolite such as kynurenic acid concentrations can provide neuroprotection against hypoxia-ischemia and NMDA lesions in neonatal rats [26]. Kynurenic acid is one of the main products of the kynurenine pathway that is produced from L-tryptophan. The neuroprotective effect of this compound was first attributed to its antagonistic action on ionotropic NMDA glutamate receptors, although other mechanisms are also involved [27]. In fact, the kynurenine metabolic pathway in human neurons was characterized to show that its association with neuroprotection depends on the expression of key metabolic enzymes [28]. For instance, the expression of picolinic carboxylase in primary and in some adult neurons leads to production of the excitotoxic quinolinic acid, whereas the lack of the enzyme in SK-N-SH cells allows the neuroprotectant picolinic acid to accumulate. Therefore, the presence or absence of a single enzyme may lead to the production of a neuroprotective or neurotoxic metabolite.

It should be noted that it appears to be safe to administer MTA exogenously to both animals and cell lines. In fact, a good safety record has already been shown in phase I studies reported in 1984 [29]. The treatment of CNS diseases requires drugs to enter the brain by crossing the BBB. Given the data from the BBB models used here, it would appear that oral administration of MTA would enhance its concentration in the brain. In conclusion, MTA appears to be a safe candidate to offer neuroprotection and to produce benefits in a variety of neurological diseases, making it potentially suitable to enter into clinical trials.

## Supporting Information

Figure S1 **No effect of MTA treatment on mossy fiber sprouting after pilocarpine-induced status epilepticus (SE).** a-c) Representative Timm-stained sections 30 days after SE from a vehicle-treated control (a) and animals that received MTA (30 mg/kg) post –SE (b) or pre-SE (c) show no difference in mossy fiber sprouting. d, e) Quantification of Timm staining density in the inner molecular layer at 30 d after SE showed no differences between groups (d, *p* = 0.84; e, *p* = 0.86; Student *t*-test). n = 4-5/condition. The data represent the mean ± SEM.
(TIF)
