# Supplementary figures and images for: Differential Neuroprotective Effects of 5′-Deoxy-5′-Methylthioadenosine
Source: PLoS One. 2014 Mar 5;9(3):e90671. doi: 10.1371/journal.pone.0090671 (PMC3944389; doi:10.1371/journal.pone.0090671)

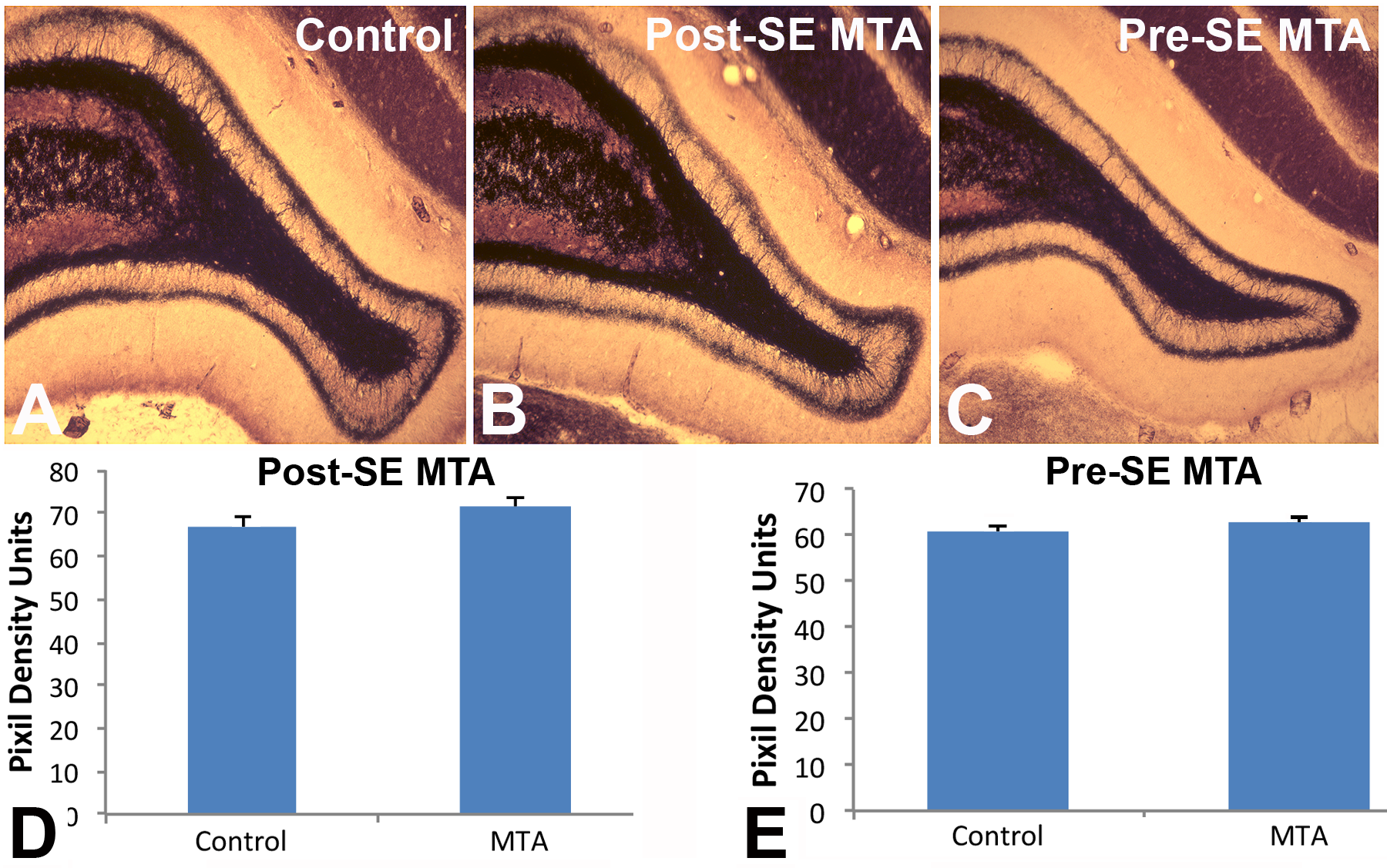

Supplement: Figure S1 — No effect of MTA treatment on mossy fiber sprouting after pilocarpine-induced status epilepticus (SE). a-c) Representative Timm-stained sections 30 days after SE from a vehicle-treated control (a) and animals that received MTA (30 mg/kg) post –SE (b) or pre-SE (c) show no difference in mossy fiber sprouting. d, e) Quantification of Timm staining density in the inner molecular layer at 30 d after SE showed no differences between groups (d, p = 0.84; e, p = 0.86; Student t-test). n = 4-5/condition. The data represent the mean ± SEM. (TIF) [file pone.0090671.s001.tif]
